# Associations between a fetal imprinted gene allele score and late pregnancy maternal glucose concentrations

**DOI:** 10.1016/j.diabet.2017.03.002

**Published:** 2017-09

**Authors:** C.J. Petry, K. Mooslehner, P. Prentice, M.G. Hayes, M. Nodzenski, D.M. Scholtens, I.A. Hughes, C.L. Acerini, K.K. Ong, W.L. Lowe, D.B. Dunger

**Affiliations:** a Department of Paediatrics, Box 116, Addenbrooke's Hospital, University of Cambridge, Hills Road, CB2 0QQ Cambridge, UK; b Division of Endocrinology, Metabolism, and Molecular Medicine, Department of Medicine, Northwestern University Feinberg School of Medicine, Chicago, IL, USA; c Division of Biostatistics, Department of Preventive Medicine, Northwestern University Feinberg School of Medicine, Chicago, IL, USA; d Medical Research Council Epidemiology Unit, University of Cambridge, Cambridge, UK; e Medical Research Laboratories, The Institute of Metabolic Science, University of Cambridge, Cambridge, UK

**Keywords:** Gestational diabetes, *KCNQ1*, Meta-analysis, Placenta, BMI, body mass index, CI, confidence interval, eQTL, expression quantitative trait locus, GDM, gestational diabetes, HAPO, Hyperglycaemia and Adverse Pregnancy Outcome, IADPSG, International Association of Diabetes in Pregnancy Study Groups, OGTT, oral glucose tolerance test, GWAS, genome wide association study, SNP, single nucleotide polymorphism, T2DM, type 2 diabetes

## Abstract

**Aim:**

We hypothesised that some of the genetic risk for gestational diabetes (GDM) is due to the fetal genome affecting maternal glucose concentrations. Previously, we found associations between fetal *IGF2* gene variants and maternal glucose concentrations in late pregnancy.

**Methods:**

In the present study, we tested associations between SNP alleles from 15 fetal imprinted genes and maternal glucose concentrations in late pregnancy in the Cambridge Baby Growth and Wellbeing cohorts (1160 DNA trios).

**Results:**

Four fetal SNP alleles with the strongest univariate associations: paternally-transmitted *IGF2* rs10770125 (*P*-value = 2 × 10^–4^) and *INS* rs2585 (*P*-value = 7 × 10^–4^), and maternally-transmitted *KCNQ1(OT1)* rs231841 (*P*-value = 1 × 10^–3^) and *KCNQ1(OT1)* rs7929804 (*P*-value = 4 × 10^–3^), were used to construct a composite fetal imprinted gene allele score which was associated with maternal glucose concentrations (*P*-value = 4.3 × 10^–6^, *n* = 981, r^2^ = 2.0%) and GDM prevalence (odds ratio per allele 1.44 (1.15, 1.80), *P*-value = 1 × 10^–3^, *n* = 89 cases and 899 controls). Meta-analysis of the associations including data from 1367 Hyperglycaemia and Adverse Pregnancy Outcome Study participants confirmed the paternally-transmitted fetal *IGF2/INS* SNP associations (rs10770125, *P*-value = 3.2 × 10^–8^, rs2585, *P*-value = 3.6 × 10^–5^) and the composite fetal imprinted gene allele score association (*P*-value = 1.3 × 10^–8^), but not the maternally-transmitted fetal *KCNQ1(OT1)* associations (rs231841, *P*-value = 0.4; rs7929804, *P*-value = 0.2).

**Conclusion:**

This study suggests that polymorphic variation in fetal imprinted genes, particularly in the *IGF2/INS* region, contribute a small but significant part to the risk of raised late pregnancy maternal glucose concentrations.

## Introduction

Familial studies suggest that the risk for a pregnant woman developing gestational diabetes (GDM) might be partially genetically mediated. However, knowledge of GDM genetics has lagged behind that of non-gravid diabetes [1], [2]. Only one genome wide association study (GWAS) focussing on GDM has been published to date [3], with another one focussing on its endophenotype, maternal glucose concentrations in pregnancy [4]. Other studies have picked candidate genes from type 2 diabetes (T2DM) GWASs to test their associations with GDM, using the assumption that the genetic architecture of GDM is very similar to that of T2DM [5], [6]. Meta-analyses have confirmed some of the associations between genetic variants and GDM [7], [8], and like with T2DM, pathway analysis of these variants shows an enrichment of insulin secretion genes [1].

Despite the known GDM-related variants, relatively little of its heritability has been explained [9]. Whilst further risk single nucleotide polymorphisms (SNPs) may emerge through the GWASs of larger populations with greater statistical power, other heritability may be explained by epistasis or copy number variants. Also, following the suggestion that the fetal genome may influence maternal pregnancy metabolism [10], we hypothesised that imprinted fetal growth genes might alter maternal glucose concentrations and GDM risk [11]. These genes are related to parent of origin effects reflecting the separate reproductive needs of each parent [12]. Paternally-expressed imprinted genes tend to enhance fetal growth, whereas maternally-expressed genes tend to reduce it, probably through changes in fetal demand and supply [13].

We first tested our hypothesis in a mouse model where *H19* genes and *Igf2* control elements (regulators of *Igf2* expression) were disrupted [14]. On day 16 of the 21.5 days of pregnancy essentially wild type mice carrying litters where half of the fetuses were *H19* knockouts had higher blood glucose concentrations than those of genetically-matched controls. These studies were followed by genotyping in humans where 4 paternally-expressed fetal *IGF2* SNPs were associated with late pregnancy maternal glucose concentrations [15]. The effect of imprinting was confirmed through lack of associations with the equivalent maternally-transmitted alleles. More recently we have found associations between various fetal imprinted gene SNP alleles and maternal blood pressure and risk of gestational hypertension [16]. Although the mechanism of how the fetal genotype can affect maternal glucose concentrations in pregnancy is unknown, we hypothesised that it involves the fetal-derived placenta through the secretion of hormones and regulatory proteins [11]. Hence in the present study, to explore the hypothesis further, we genotyped SNPs in several fetal imprinted genes that are expressed in the placenta at some stage of fetal development [17]. We tested their associations with late pregnancy maternal glucose concentrations in our Cambridge Baby Growth and Wellbeing cohorts, initially focussing on just paternally-expressed fetal genes because they are independent of confounding by the maternal genotype. We constructed a composite fetal imprinted gene allele score to estimate the extent to which polymorphic variations in these genes are associated with variance in late pregnancy maternal glucose concentrations and GDM in our cohorts. We then we used data from Hyperglycaemia and Adverse Pregnancy Outcome (HAPO) Study participants [18] to perform replication testing and meta-analyses of the associations.

## Material and methods

### Cohort 1: Cambridge Baby Growth Study

The prospective, longitudinal Cambridge Baby Growth Study recruited mothers (and their partners and offspring) attending early pregnancy ultrasound clinics at the Rosie Maternity Hospital, Cambridge, UK. between the years 2001–2009 [19]. At around 28 weeks of gestation, the mothers underwent a 75 g oral glucose tolerance test (OGTT) after fasting overnight. Venous blood was collected just prior to and 60 min after the consumption of the glucose load for the measurement of plasma glucose and insulin concentrations. In total, 845 DNA trios were collected from the families of 1074 mothers recruited to the study for whom OGTT data were available. Blood and/or mouth swab samples for DNA extraction were collected from the father and the offspring after birth. In this cohort 96.9% of the offspring were White, 0.8% were mixed race, 0.6% were Black (African or Caribbean), 0.8% were Oriental and 0.9% were Indo-Asian. Using International Association of Diabetes in Pregnancy Study Groups (IADPSG) thresholds [20] the GDM prevalence was 10.2%.

### Cohort 2: Cambridge Wellbeing Study

The Cambridge Wellbeing Study is a retrospective study of mothers, fathers and children where the mother had delivered a full-term, singleton baby at the Rosie Maternity Hospital, Cambridge, UK between the years 1999–2000 [15]. Routinely collected clinical data were available on offspring birth weight and mother's whole blood glucose levels measured 60 min after the oral consumption of 50 g glucose at 27–29 weeks of gestation. Exclusion criteria were pre-existing maternal hypertension and diabetes treatment during early pregnancy. All the offspring were White. We sought permission from the mother's General Practitioner to approach the family to collect their DNA sample by mouth swab kits. In total 315 DNA trios were collected out of 563 women who consented. Using IADPSG thresholds [20] the GDM prevalence was 8.2%.

### Cohort 3: Hyperglycaemia and Adverse Pregnancy Outcome Study

HAPO is a large multi-national prospective study of pregnancy that has been described previously, including its exclusion criteria [18], [21]. Pregnant study participants, recruited early in gestation between the years 2000–2006, underwent a 75 g OGTT as close to 28 weeks of gestation as possible. Glucose concentrations were measured centrally. Those women with plasma glucose concentrations > 5.8 mmol/L (fasting) or 11.1 mmol/L (two hours into the OGTT), 1.7% of the total population, were excluded from the study due to having overt diabetes. DNA samples from 1424 mother and baby study participants of European ancestry were used for a GWAS of maternal glycaemic and new-born anthropometric traits [4]. Using IADPSG thresholds [20], the GDM prevalence was 14.8%.

### Ethical approval

The Cambridge Baby Growth and Wellbeing Studies were approved by the local ethics committee, Addenbrooke's Hospital, Cambridge, UK. In the HAPO Study, the protocol was approved by each field centre's local institutional review board. Written informed consent was obtained from the parents in each of the cohorts studied, including consent for inclusion of their infants in the study.

### Biochemical measurements

Blood glucose concentrations were measured using a routine glucose oxidase-based method. Maternal plasma insulin concentrations were measured using a DSL ELISA kit (London, UK) according to the manufacturer's instructions.

### Gene selection and genetics

For the Cambridge Baby Growth and Wellbeing Studies genomic DNA was extracted from blood samples or mouth swabs using an Autopure LS Machine (Qiagen Ltd., Crawley, UK). The 15 imprinted genes that were studied (*DLK1, FAM99A, GNAS, GRB10, IGF2, INS, KCNQ1OT1, MEST, NNAT, PEG3, PEG10, PLAGL1, SGCE, SNRPN, ZIM2*) were chosen because they are all paternally- and placentally-expressed at some stage of development [17]. The variants that were genotyped were haplotype tag SNPs covering the gene and 20 kb either side of it, identified by Tagger (r^2^ > 0.8 and minor allele frequency > 0.2) from the Centre d’Etude du Polymorphisme Humain population of HapMap Project Build 36 using Haploview [22] (Table S1; see supplementary material associated with this article online). The one exception to this was 11 tagging *IGF2* SNPs, which were identified by Rodríguez et al. [23]. The DNA samples were genotyped using Kompetitive Allele specific PCR assays, which are competitive allele specific PCR SNP genotyping assays using fluorescence resonance energy transfer quencher cassette oligonucleotides (designed and performed by LGC Genomics, Hoddesdon, UK). The genotypes that were used in this study were consistent with Hardy Weinberg equilibrium (*P *> 0.05 using the χ^2^ test) and had a repeat genotyping discordancy rate of < 1.0%.

HAPO DNA samples were genotyped using the Illumina Human 610 Quad v1 B SNP array (Illumina Inc., San Diego, USA) and additional SNPs were imputed using BEAGLE [24]. For the present study, the SNP genotypes that were tested from the HAPO population were those that were used in the composite fetal allele imprinted gene allele score (namely paternally-transmitted fetal *IGF2* rs10770125 and *INS* rs2585, and maternally-transmitted fetal *KCNQ1OT1* rs231841 and *KCNQ1OT1* rs7929804).

### Placental *KCNQ1(OT1)* gene expression

*KCNQ1* and *KCNQ1OT1* expression were measured in term placentas by RT-qPCR with *YWHAZ*, *TOP1* and *UBC* used as reference genes [25] (see the detailed methodology in Appendix; see supplementary material associated with this article online). Expression was then related to the fetal *KCNQ1OT1* SNP alleles that were associated with maternal glucose concentrations.

### Statistical analyses and composite score formulation

With different glucose loads used in the two Cambridge cohorts, post-load maternal glucose responses were standardised by calculating z-scores separately in each cohort (using the mean and standard deviation for glucose concentrations from all the women in each cohort), and then analysed as a single group (with more statistical power) of 1160 family DNA trios with maternal glucose z-scores [15]. The Cambridge Wellbeing Study glucose concentration z-scores were also used (along with the mean and standard deviation glucose concentrations from the Cambridge Baby Growth Study) to convert the Wellbeing Study glucose concentrations to the equivalent glucose concentrations in the Cambridge Baby Growth Study. This approach, along with assuming a minor allele frequency for each SNP of 0.3 and consistency with Hardy Weinberg equilibrium, meant that there was 90% statistical power to be able to detect a difference of 0.23 maternal glucose z-scores (equivalent to 0.4 mmol/L) (α = 0.05).

SNP genotypes from both parents and their child were used to infer parental transmission (Table S2(a); see supplementary material associated with this article online). In HAPO, where paternal genotypes were unavailable, parental transmission was inferred as per Table S2(b) (see supplementary material associated with this article online).

Each parentally-transmitted fetal SNP allele was tested for association with the 60 min post-load maternal glucose concentration z-score in an unadjusted linear regression model (i.e. paternally- and maternally-transmitted SNP alleles were tested separately). Where needed robust regression was used where the standard errors were estimated using the Huber-White sandwich technique to overcome minor concerns about failures to meet regression model assumptions. The *P*-values for the associations were recorded and ranked. The composite fetal imprinted gene allele score was then constructed starting with the association with the lowest *P*-value, and then adding one further fetal SNP allele at a time in gradually increasing order of *P*-values [16]. Allele scores from more than one variant per gene were allowed if the two variants were not in linkage disequilibrium (defined when the study was designed as r^2^ > 0.8) in the Thousand Genomes Project [26]. For each SNP that was included in the allele score, where the fetal SNP allele was known a score of 1 was added if it was the one that was associated with higher glucose concentrations and 0 if it was the one associated with lower concentrations. In cases where the allele was missing (due to genotyping error or uninformative trio genotypes) the combined frequency amongst both cohorts of the glucose increasing allele was added to the score. After each new allele was added to the gene score associations with the 60 min post-load maternal glucose concentration z-score was retested in a linear regression model. This process was continued, adding scores from one SNP allele at a time, whilst the adjusted r^2^ of the linear regression model was increasing. Once the r^2^ of the model reduced (due to greater noise brought about by the addition of an allelic score from a SNP that was more weakly-associated with the maternal glucose concentrations) this process was halted and the previous allele score was adopted as the final score (an unweighted estimated allele count). To remove potential overfitting due to linkage disequilibrium further allele scores were calculated removing effects due to each of the individual *IGF2* SNP alleles in the full allele score. Associations with GDM were tested using logistic regression, both unadjusted and adjusted for pre-pregnancy maternal body mass index (BMI) and age.

The meta-analysis was performed using correlation coefficients of the associations between the fetal alleles and week 28 maternal glucose concentrations 60 min after a glucose load. For the Cambridge cohorts’ correlation, coefficients were available to us. For the HAPO Study participants standardised β-coefficients were available to us and correlation coefficients were estimated from these using the method of Peterson and Brown [27]. The meta-analysis used the DerSimonian-Laird approach [28] for random effects models to allow for any potential heterogeneity, implemented into the R package Metacor version 1.0-2 [29]. Meta-analysis heterogeneity was assessed using Medcalc version 17.2 (Ostend, Belgium).

Correction for multiple testing was considered unnecessary as only one primary association was tested (i.e. the allelic score with the maternal glucose z-scores). All other associations were considered secondary and a *P*-value of < 0.05 was considered statistically significant. Unless otherwise stated data are presented as mean (95% confidence interval (CI)). Effect sizes are presented as Cohen's d (where there are two groups being compared) or adjusted r^2^ values (where there are more than two groups being compared). All statistical analyses were performed using either Stata version 13 (StataCorp LP, College Station, Texas, USA) or R version 3.2.2 [30].

## Results

### Associations with maternal glucose concentration Z-scores and gestational diabetes in the Cambridge baby growth and wellbeing studies

**Fig. 1:**
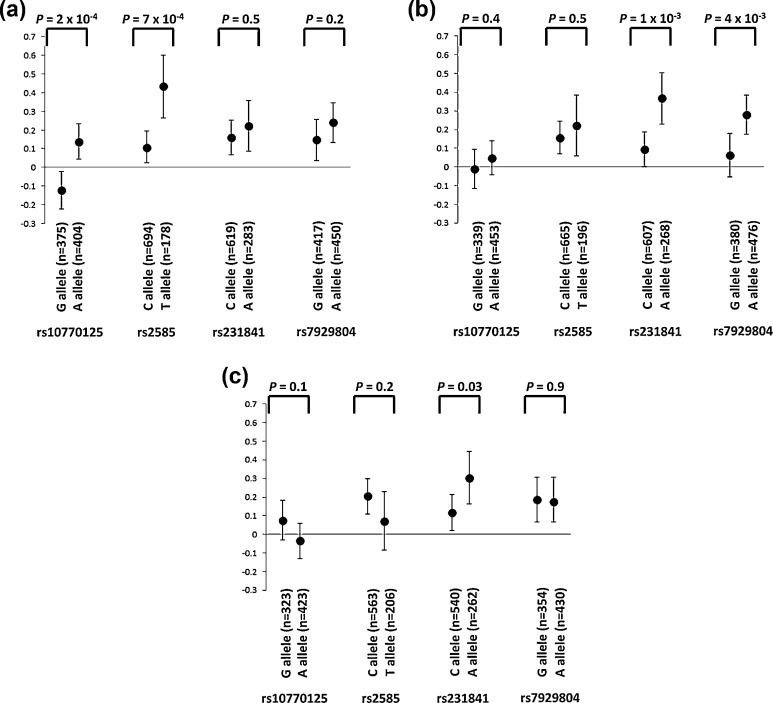
Associations between maternal glucose concentration z-scores one hour after the oral consumption of a glucose load and alleles from the 4 SNPs that are used in the composite fetal imprinted gene allele score: a: the paternally-transmitted fetal allele; b: the maternally-transmitted fetal allele; c: the maternal allele that was not transmitted to the fetus. Data are mean (95 % confidence interval).

The ten strongest unadjusted associations between fetal imprinted gene alleles and maternal 60 min glucose concentration z-scores are shown in Table 1, including 4 *IGF2* SNPs previously reported [15]. All other SNP alleles were associated at *P *> 0.01. The composite fetal imprinted gene allele score comprised the scores from the genotypes of the first four variants listed in Table 1 (i.e. paternally-inherited rs10770125 and rs2585, and maternally-inherited rs231841 and rs7929804). No associations with unadjusted maternal glucose concentrations were found with the same alleles transmitted from the other parents (Fig. 1a and b). For rs231841 and rs7929804, there were stronger associations observed with the transmitted rather than untransmitted alleles (correlation coefficients of 0.10 and 0.07, respectively, for rs231841, and 0.09 and 0 for rs7929804; Fig. 1b and c).

**Table 1:** The ten associations between fetal imprinted gene SNP alleles and maternal glucose concentration z-scores one after the oral consumption of a glucose load in the Cambridge Baby Growth and Wellbeing Studies, with the lowest *P*-values in linear regression models. Also shown is the *P*-values of the associations of maternal glucose concentration z-scores with the equivalent maternal genotypes.

Gene	SNP	Parental transmission	Non-risk allele	Risk allele	Fetal allele *P*-value	Effect size	Maternal genotype *P* -value	Fetal allele *P*-value in white participants only (*n* = 672∼760)
*IGF2, INS*	rs10770125	Paternal	–0.122 (–0.222, –0.023) (*n* = 375)	0.138 (0.042, 0.233) (*n* = 404)	0.0002	0.26	0.7	0.0004
*IGF2, INS*	rs2585	Paternal	0.107 (0.022, 0.193) (*n* = 694)	0.433 (0.265, 0.602) (*n* = 178)	0.0007	0.29	0.6	0.005
*KCNQ1OT1*	rs231841	Maternal	0.093 (0.001, 0.185) (*n* = 607)	0.367 (0.229, 0.505) (*n* = 268)	0.001	0.24	3.1 × 10^–4^	0.03
*KCNQ1OT1*	rs7929804	Maternal	0.052 (–0.055, 0.158) (*n* = 377)	0.279 (0.170, 0.387) (*n* = 476)	0.004	0.20	0.2	0.07
*KCNQ1OT1*	rs231352	Maternal	0.051 (–0.052, 0.155) (*n* = 425)	0.281 (0.165, 0.397) (*n* = 441)	0.004	0.20	8.5 × 10^–5^	0.1
*KCNQ1OT1*	rs231361	Maternal	0.121 (0.038, 0.203) (*n* = 741)	0.378 (0.217, 0.538) (*n* = 195)	0.005	0.22	1.8 × 10^–4^	0.008
*IGF2, INS*	rs7924316	Paternal	–0.087 (–0.181, 0.007) (*n* = 423)	0.109 (0.004, 0.214) (*n* = 337)	0.006	0.20	0.8	0.004
*IGF2*	rs6578987	Paternal	–0.060 (–0.138, 0.018) (*n* = 608)	0.156 (0.015, 0.298) (*n* = 105)	0.009	0.22	1.0	0.02
*IGF2*	rs680	Paternal	–0.065 (–0.142, 0.013) (*n* = 613)	0.144 (0.001, 0.288) (*n* = 180)	0.01	0.21	0.9	0.02
*IGF2*	rs4320932	Paternal	–0.026 (–0.099, 0.046) (*n* = 719)	0.224 (0.046, 0.403) (*n* = 118)	0.01	0.25	0.8	0.01

Data shown are mean (95% confidence interval) z-scores. Effect sizes are Cohen's d.

The composite fetal imprinted gene allele score was strongly associated with unadjusted 60 min maternal glucose concentrations (*P *= 4.3 × 10^–6^, *n* = 981). The score explained 2.0% of the maternal glucose concentration variance and each increase of one in the score was associated with an increase in maternal 60 min glucose concentration z-score of 0.15 (≈0.26 mmol/L). Significant associations were seen in each of the two cohorts separately (Cambridge Baby Growth Study: *P *= 4.8 × 10^–4^, adjusted r^2^ = 1.7%, *n* = 664; Wellbeing Study: *P *= 3.0 × 10^–3^, adjusted r^2^ = 2.5%, *n* = 317). Table S3 (Table S3; see supplementary material associated with this article online) shows the *P*-values for the individual SNPs in each cohort. Table S4 (Table S4; see supplementary material associated with this article online) shows the *P*-values for the association with the composite fetal imprinted gene allele score minus effects of paternally-transmitted fetal *IGF2* rs10770125 and rs2585 individually. The full composite fetal imprinted gene allele score was also associated with unadjusted GDM prevalence (Table 2). The mean (95% CI) allele scores per group were: controls 1.53 (1.47, 1.59) (*n* = 899) v. cases 1.87 (1.68, 2.07) (*n* = 89).

**Table 2:** Associations between the composite fetal imprinted gene allele score and the prevalence of GDM.

Cohorts	Statistical model	Odds ratio per allele	*P*-value	Pseudo r^2^ (%)	Number of observations
Cambridge Baby Growth Study & Cambridge Wellbeing Study combined	Unadjusted	1.44 (1.15, 1.80)	1.3 × 10^–3^	1.7	988
Cambridge Baby Growth Study & Cambridge Wellbeing Study combined	Adjusted for maternal age	1.42 (1.14, 1.78)	1.9 × 10^–3^	1.6	956
Cambridge Baby Growth Study & Cambridge Wellbeing Study combined	Adjusted for pre-pregnancy maternal BMI	1.38 (1.02, 1.86)	0.035	4.4	556
Cambridge Baby Growth Study & Cambridge Wellbeing Study combined	Adjusted for maternal age and pre-pregnancy maternal BMI	1.37 (1.01, 1.84)	0.041	4.4	534
Cambridge Baby Growth Study	Unadjusted	1.42 (1.09, 1.86)	0.011	1.5	671
Cambridge Wellbeing Study	Unadjusted	1.50 (1.00, 2.24)	0.049	2.2	317

Odds ratios are mean (95% confidence interval).

The composite fetal imprinted gene allele score was also associated with the unadjusted fasting blood glucose concentrations (*P *= 0.03; *n* = 671 in the Cambridge Baby Growth Study). The score explained 0.6% of the maternal fasting glucose concentration variance and for each increase of the gene score by one there was an associated increase in maternal glucose concentration z-scores of 0.02 (≈0.04 mmol/L). The composite allele score was also associated with the unadjusted maternal 60 min plasma insulin concentrations (*P *= 0.03; adjusted r^2^ = 0.4%, *n* = 706) and with the unadjusted insulin increment after the glucose load (*P *= 0.03; adjusted r^2^ = 0.5%, *n* = 706).

### Replication in HAPO and meta-analysis

**Fig. 2:**
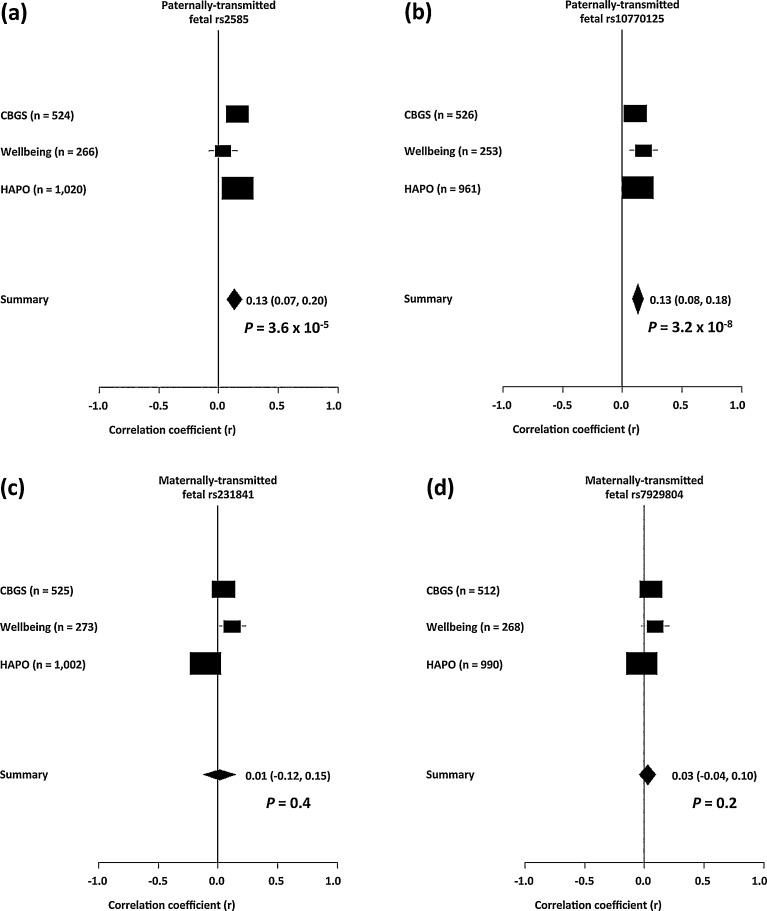
Forest plots of the random effects meta-analysis of the associations of the 4 SNPs that are used in the composite fetal imprinted gene allele score with maternal glucose concentrations 60 min into an OGTT showing contributing results from the Cambridge Baby Growth Study, the Cambridge Wellbeing Study and the HAPO Study participants with European ancestry: a: paternally-transmitted fetal rs2585; b: paternally-transmitted fetal rs10770125; c: maternally-transmitted fetal rs231841; d: maternally-transmitted fetal rs7929804.

**Fig. 3:**
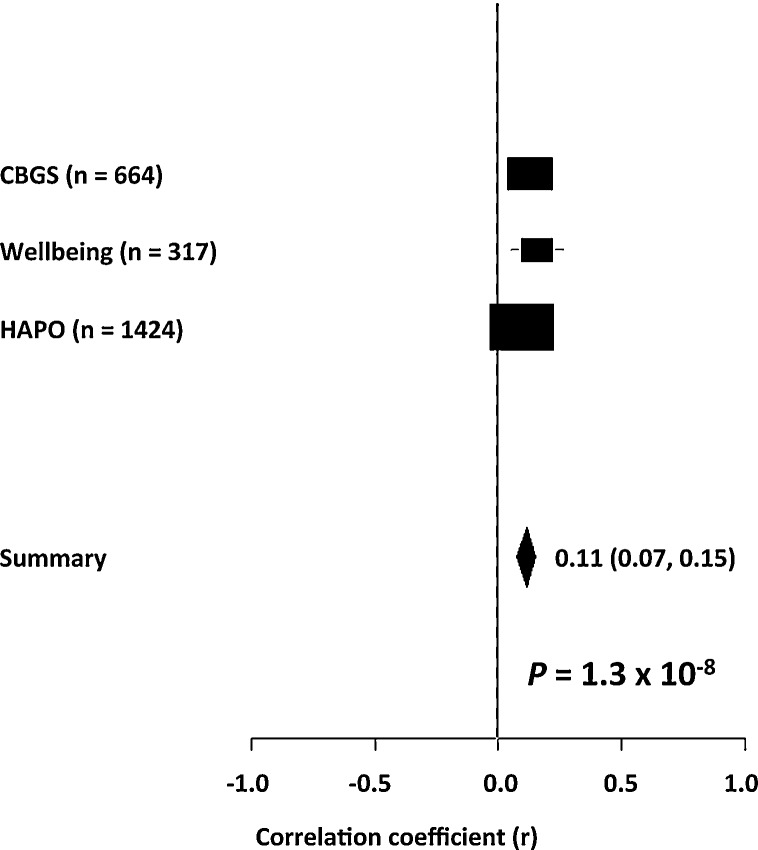
Forest plot of the random effects meta-analysis of the associations of the composite fetal imprinted gene allele score with maternal glucose concentrations 60 min into an OGTT showing contributing results from the Cambridge Baby Growth Study, the Cambridge Wellbeing Study and the HAPO Study participants with European ancestry.

None of the four fetal SNPs used in the composite allele score or the composite allele score itself were significantly associated with unadjusted maternal glucose concentrations 60 min into the OGTT in the HAPO Study participants (Table 3). However, two of the univariate associations and the composite fetal imprinted gene allele score association were unidirectional with those from the Cambridge cohorts, so that in the meta-analysis the inclusion of the HAPO Study data strengthened the associations with maternal 60 min glucose concentrations found in the Cambridge cohorts for the paternally-transmitted fetal SNP alleles from the *INS*/*IGF2* region, rs2585 and rs10770125 (*P *= 3.6 × 10^–5^ and *P *= 3.2 × 10^–8^, respectively) (Fig. 2). In contrast the meta-analysis showed no significant associations with the maternally-transmitted fetal SNP alleles from the *KCNQ1OT1* region, rs231841 and rs7929804 (*P *= 0.4 and *P *= 0.2, respectively). The composite fetal imprinted gene allele score association with maternal 60 min glucose concentrations found in the Cambridge cohorts was strengthened in the meta-analysis (*P *= 1.3 × 10^–8^) (Fig. 3). None of the meta-analyses had significant heterogeneity (Table S5; see supplementary material associated with this article online).

**Table 3:** Associations between the four fetal SNP alleles used in the composite fetal imprinted gene allele score, and the score itself, and maternal glucose concentrations one hour after the oral consumption of a 75 g glucose load in HAPO Study participants with European ancestry.

Fetal SNP	Directly genotyped or imputed?	Imputation quality	Parental transmission	Risk allele	*n*	Standardised β-Coefficient	Standard error of the β-Coefficient	*P*-value
rs2585	Imputed	0.975	Paternal	T	1020	0.112	0.112	0.32
rs231841	Genotyped	N/A	Maternal	A	1002	–0.106	0.119	0.37
rs10770125	Imputed	0.96	Paternal	A	961	0.080	0.104	0.44
rs7929804	Genotyped	N/A	Maternal	A	990	–0.024	0.104	0.81
Composite fetal imprinted gene allele score	N/A	N/A	Both	N/A	1424	0.048	0.047	0.31

N/A: not applicable.

### Placental *KCNQ1(OT1)* gene expression

Maternally-transmitted fetal rs231841 allele was associated with placental *KCNQ1* expression at birth adjusted for the expression of the panel of reference genes (*n* = 21 samples; *P *= 0.04) in the Cambridge Baby Growth Study but not with placental *KCNQ1OT1* expression (*n* = 20; *P *= 0.2) (Table S6; see supplementary material associated with this article online). Maternally-transmitted fetal rs7929804 was not associated with the expression of either gene (*n* = 20 for both; *P *= 0.9 and 0.3, respectively).

## Discussion

In this study, we developed a composite fetal imprinted gene allele score that was strongly associated with late pregnancy maternal glucose concentrations and GDM in the two independent Cambridge cohorts. Significance of the association was not reached in HAPO Study participants with European ancestry, although the association was unidirectional with the associations in the Cambridge cohorts and genome wide significance was reached by meta-analysis. The lack of significant replication of associations outside of the meta-analysis for the individual SNP alleles and the composite score is perhaps not surprising given that the HAPO Study excluded those with the highest glucose concentrations and participants with European ancestry were recruited from 4 different sites in the USA, 2 in the UK, 2 in Australia and 1 in Canada [4] whereas both Cambridge cohorts were recruited via the same maternity hospital in the UK and so may be more homogeneous. The association between maternal glucose concentrations and the composite fetal imprinted gene allele score in the meta-analysis suggests that the fetal genes that were tested, with the *INS/IGF2* region in particular, may make a small but significant contribution to the maternal glucose concentrations in pregnancy. This expands our previous finding of 4 paternally-transmitted fetal *IGF2* SNP alleles that were also associated with maternal glucose concentrations in the two Cambridge cohorts [15]. In our meta-analysis, the association of one of these *IGF2* SNP alleles (rs10770125) with maternal glucose concentrations reached genome wide significance, the first fetal SNP allele to do so.

Although variants were tested from 15 different paternally-expressed imprinted genes, the ten variants most strongly associated in our data were found in only two fetal regions, *IGF2/INS* and *KCNQ1/KCNQ1OT1*. Although another 4 loci from the 142 fetal imprinted gene SNPs tested were nominally associated with maternal glucose concentrations (0.01 <* P *< 0.05; data not shown) their associations did not contribute to the composite score. Each of the two fetal genetic regions represented in the composite score contributed two variants. The *IGF2* and *INS* genes are adjacent on chromosome 11p15, separated by only 15 kb. Insulin is not a classically imprinted gene in humans but is imprinted and paternally-expressed in the embryonic human yolk sac [31] and parent of origin associations and linkage have previously been found with T2DM [32], whereas *IGF2* is a key imprinted gene for fetal growth. Of the two SNPs that contribute to the construction of the composite score rs10770125 is intronic for *IGF2* but missense for the *INS-IGF2* read through transcript, coding for a leucine to proline substitution [15], and is also in linkage disequilibrium with rs1003483, an expression quantitative trait locus (eQTL) for *IGF2.* Similarly, rs2585 is in linkage disequilibrium with rs11042594 which is also an eQTL for *IGF2.* Although there might be a degree of linkage disequilibrium between *IGF2* rs10770125 and rs2585 (r^2^ = 0.28 and D’ = 0.92 in our population) if the effects of either paternally-transmitted allele were removed from the gene score the associations with maternal glucose concentrations were still apparent, suggesting that the association with the composite gene score does not arise from overfitting. The other two fetal SNP alleles contributing to the composite score are in the *KCNQ1OT1* region and are maternally-transmitted, even though this study was established to investigate associations with paternally-expressed genes. However, the genetic region including the *KCNQ1OT1* gene and 20 kb either side of it would encompass part of the maternally-expressed *KCNQ1* imprinted gene and therefore the SNPs might reflect that gene as well. The placental expression data would also suggest that the primary association is with *KCNQ1* rather than *KCNQ1OT1 per se*, at least for rs231841*.* Interestingly several studies have previously found associations between genetic variation in *KCNQ1* and GDM [7], [33], [34], [35], [36], [37]. Of the two SNPs used in the composite score, rs231841 shows a degree of linkage disequilibrium (r^2^ = 0.75, D’ = 0.88) in the Thousand Genome Project [26] with rs231353 which itself is associated with T2DM [38]. The other SNP rs7929804 is in linkage disequilibrium with rs10766218 (r^2^ = 0.8, D’ = 1) [26], which is an eQTL for *KCNQ1*, whose expression is associated with insulin secretion *in vitro* [39]. This is consistent with the association that we found with the insulin increment after the glucose load. The associations with these fetal alleles therefore appear plausible.

The fact that in the meta-analysis of the univariate fetal SNP allele associations the *KCNQ1(OT1)* associations were not significant raises the possibility that their associations in the Cambridge cohorts are confounded by the maternal ones. Indeed, unlike associations with paternally-transmitted fetal alleles, associations with maternally-transmitted fetal alleles are extremely difficult to distinguish from maternal genotype effects. Maternal allelic transmission to the fetus obviously originates from the maternal genotype and is therefore not biologically independent of it. Indeed, for rs231841 there is evidence in the Cambridge cohorts for a direct maternal genotype association with their glucose concentrations, its p-value being lower than that with the maternally-transmitted fetal allele. However, there is possibly an additional fetal genetic effect because the association with the maternally-transmitted fetal allele always has a lower p-value than that with the untransmitted maternal allele and the association of the fetal genotype with placental *KCNQ1* expression is stronger than that with the maternal genotype. We must be cautious about these interpretations, however, given the lack of replication of these univariate associations in HAPO; it remains possible that all the fetal associations with maternal GDM-related phenotype are mediated through *IGF2* once confounders have been fully accounted for*.*

Although the associations between the maternal glucose concentrations and the composite fetal imprinted gene allele score appear robust given the meta-analysis, with their lack of heterogeneity, the study does have limitations in addition to those outlined previously [15]. Firstly, the analysis used to construct the composite fetal imprinted gene allele score is *post-hoc* in nature. However, we set out to construct it this way due to the lack of established fetal imprinted gene allele associations with maternal glucose concentrations in pregnancy. This score is now available to be tested in other cohorts. Another limitation is the relatively modest size of the Cambridge cohorts, although this is mitigated somewhat by the meta-analysis with HAPO. Also, the association between maternal glucose concentrations and the composite fetal imprinted gene allele score reached significance in two independent cohorts, but the individual SNPs that contribute to that score needed the two independent cohorts to be analysed together to have sufficient statistical power for their associations to reach statistical significance. For a discovery set of SNPs used to create a composite score that is acceptable, however. A final limitation is that the glucose concentrations from the Cambridge Wellbeing Study had to be transformed to equivalent values from the Cambridge Baby Growth Study for comparison of them to IADPSG guidelines for GDM. This assumes that maternal glucose concentrations one hour into the OGTT are completely consistent with those from a 50 g glucose load where the mother was not necessarily fasted (although a number would have been). However, the two cohorts were drawn from essentially the same local population, and high glucose concentrations after the 50 g load are used to predict those mothers who will have high glucose concentrations after fasting and the 75 g oral glucose load, so the modelling is not unrealistic. In addition, the SNP genotype associations were very similar in each separate Cambridge cohort.

In summary, we have developed a composite fetal imprinted gene allele score that is associated with maternal glucose concentrations and GDM prevalence in two Cambridge birth cohorts, and genome wide significance is reached when the association with maternal glucose concentrations is analysed by meta-analysis using data from HAPO Study participants with European ancestry. The method used to develop the composite score bypasses the problem of certain DNA trio genotypes being uninformative if all three members of that family are heterozygous. It can be used with cohorts where there are DNA samples available from the offspring and only one of the parents, as demonstrated by the results from HAPO, despite there being a higher proportion of uninformative SNPs (and therefore probably wider confidence intervals) because only two rather than three samples need to be heterozygous for this. By meta-analysis of our univariate associations with maternal glucose concentrations, we have also shown for the first time that the association one of paternally-expressed fetal *IGF2* alleles with late pregnancy maternal glucose concentrations is significant at the genome wide level. So far, with the limited number of imprinted genes that we tested, the contribution of the fetal genome to variation in maternal glucose concentrations appears to be small although highly statistically significant. In combination with maternal risk genotypes, these fetal alleles may increase the risk of maternal GDM.

## Disclosure of interest

The authors declare that they have no competing interest.
