# Supplementary material for: Associations between a fetal imprinted gene allele score and late pregnancy maternal glucose concentrations
Source: Diabetes Metab. 2017 Sep;43(4):323–31. doi: 10.1016/j.diabet.2017.03.002 (PMC5507297; doi:10.1016/j.diabet.2017.03.002)
Supplement: Supplementary file 1 [file mmc1.docx]

**Table S1:** The SNPs that were genotyped in the Cambridge Baby Growth and Wellbeing Studies as part of this study, along with their minor allele frequencies in the children.

| Gene | No. of SNPs | Single nucleotide polymorphisms |
| --- | --- | --- |
| *DLK1* | 5 | rs12147008 (0.21), rs7155375 (0.34), rs10139403 (0.29), rs1802710 (0.48), rs7147586 (0.29) |
| *FAM99A* | 7 | rs4752779 (0.36), rs4752781 (0.41), rs11600502 (0.42), rs10839220 (0.50), rs11607954 (0.47), rs1489945 (0.50), rs7131362 (0.41) |
| *GNAS* | 20 | rs6123832 (0.36), rs965808 (0.23), rs6128441 (0.44), rs6026561 (0.36), rs6026560 (0.19), rs12625436 (0.40), rs4810148 (0.35), rs7271854 (0.41), rs6100260 (0.26), rs6026576 (0.36), rs6128461 (0.36), rs6123837 (N/A), rs13831 (0.27), rs234630 (0.28), rs919196 (0.18), rs3730168 (0.31), rs919197 (0.48), rs7121 (0.48), rs234623 (0.49), rs234621 (0.29) |
| *GRB10* | 17 | rs4245555 (0.44), rs12669770 (0.34), rs7802879 (0.19), rs7777754 (0.27), rs2715129 (0.20), rs3807549 (0.33), rs2299150 (0.30), rs11769934 (0.45), rs2237441 (0.45), rs1468450 (0.38), rs6948959 (0.24), rs980716 (0.21), rs2715116 (0.46), rs737292 (0.32), rs7794604 (0.42), rs17133917 (0.44), rs2237450 (N/A) |
| *IGF2** | 17 | rs6578987 (0.28), *rs734351 (0.36), *rs4341514 (0.37), rs680 (0.27), *rs3741212 (0.33), *rs11603378 (0.25), *rs3213216 (0.36), *rs10770125 (0.48), *rs3741205 (0.45), *rs3741206 (0.34), rs1004446 (0.38), *rs4320932 (0.18), *rs3842752 (0.22), *rs7924316 (0.46), *rs7483056 (0.47), *rs6356 (0.36), *rs10743152 (0.38) |
| *INS** | 16 | *rs734351 (0.36), *rs4341514 (0.37), rs2585 (0.25), *rs3741212 (0.33), *rs11603378 (0.25), *rs3213216 (0.36), *rs10770125 (0.48), *rs3741205 (0.45), *rs3741206 (0.34), *rs4320932 (0.18), rs3741211 (0.37), *rs3842752 (0.22), *rs7924316 (0.46), *rs7483056 (0.47), *rs6356 (0.36), *rs10743152 (0.38) |
| *KCNQ1OT1/(KCNQ1)* | 14 | rs231352 (0.49), rs463924 (0.30), rs4930005 (0.26), rs231841 (0.34), rs10832514 (0.32), rs756852 (0.39), rs7128926 (0.21), rs10832430 (0.27), rs231361 (0.24), rs760419 (0.46), rs9666537 (0.27), rs7929804 (0.46), rs10766218 (0.41), rs6578283 (0.30) |
| *MEST* | 6 | rs12706933 (0.37), rs13234660 (0.29), rs765205 (0.48), rs13225903 (0.40), rs1047456 (0.29), rs10954272 (0.23) |
| *NNAT* | 3 | rs6066671 (0.41), rs12481150 (0.28), rs6019103 (0.42) |
| *PEG3** | 8 | rs17207579 (0.32), rs11084466 (0.46), *rs2079877 (0.35), *rs7256072 (0.45), *rs382592 (0.45), rs758752 (0.22), rs7248098 (0.23), *rs1860565 (0.28) |
| *PEG10* | 5 | rs1568214 (0.40), rs13073 (0.27), rs1533831 (0.29), rs6948099 (0.37), rs6465422 (0.24) |
| *PLAGL1/HYMAI* | 20 | rs12528289 (N/A), rs9484833 (0.45), rs1884087 (0.42), rs10499846 (0.06), rs2268440 (0.41), rs6930726 (0.46), rs7770529 (0.39), rs2268443 (0.28), rs2876578 (0.29), rs9373409 (0.45), rs2268445 (0.22), rs17073273 (0.30), rs9403542 (0.34), rs9484836 (0.18), rs6570599 (0.29), rs2328537 (0.44), rs9321957 (0.36), rs6937128 (0.24), rs9767615 (0.48), rs2207851 (0.39) |
| *SGCE* | 2 | rs1879854 (0.27), rs7778237 (0.40) |
| *SNRPN* | 12 | rs7164801 (0.40), rs17114774 (0.33), rs7171320 (0.43), rs1453556 (0.35), rs4028398 (0.33), rs1982981 (0.27), rs736008 (0.41), rs9806682 (0.36), rs7164989 (0.45), rs8042562 (0.48), rs12592279 (0.24), rs8029318 (0.20) |
| *ZIM2** | 8 | *rs2079877 (0.35), rs12981689 (0.32), rs1476830 (0.46), *rs7256072 (0.45), *rs382592 (0.45), *rs1860565 (0.28), rs2286751 (N/A), rs10401139 (0.23) |

Genes are named according to their HUGO (Human Genome Organisation) abbreviation.

* indicates that some of the SNPs are shared with another gene (*IGF2* with *INS* and *PEG3* with *ZIM2*). A total of 142 SNPs were genotyped. The following SNP genotypes (labelled as N/A) failed as either the assay was unsuitable or the genotypes did not pass the quality control procedures: (*GNAS*) rs6123837, (*GRB10*) rs2237450, (*PLAGL1/HYMAI*) rs12528289, (*ZIM2*) rs2286751, so their minor allele frequencies cannot be presented.
