# Supplementary material for: Associations between a fetal imprinted gene allele score and late pregnancy maternal glucose concentrations
Source: Diabetes Metab. 2017 Sep;43(4):323–31. doi: 10.1016/j.diabet.2017.03.002 (PMC5507297; doi:10.1016/j.diabet.2017.03.002)
Supplement: Supplementary file 2 [file mmc2.docx]

**Table S2:** Imputation table of parentally transmitted fetal SNP alleles from a SNP consisting of various possible combinations of major allele “A” and minor allele “a”:

- (a) where there are father’s, mother’s and fetus’ genotypes available (i.e. in the Cambridge Baby Growth Study and the Cambridge Wellbeing Study) [15] and

- (b) where there are only mother’s and fetus’ genotypes available (i.e. in the HAPO Study).

(a)

| **Paternal**  **Genotype** | **Maternal**  **Genotype** | **Fetal**  **Genotype** | **Paternally-transmitted**  **Allele** | **Maternally-transmitted**  **Allele** |
| --- | --- | --- | --- | --- |
| A/A | A/A | A/A | A | A |
| A/A | A/a | A/A | A | A |
| A/A | A/a | A/a | A | a |
| A/A | a/a | A/a | A | a |
| A/a | A/A | A/A | A | A |
| A/a | A/A | A/a | a | A |
| A/a | A/a | A/A | A | A |
| A/a | A/a | A/a | uninformative | uninformative |
| A/a | A/a | a/a | a | a |
| A/a | a/a | A/a | A | a |
| A/a | a/a | a/a | a | a |
| a/a | A/A | A/a | a | A |
| a/a | A/a | A/a | a | A |
| a/a | A/a | a/a | a | a |
| a/a | a/a | a/a | a | a |

(b)

| **Maternal**  **Genotype** | **Fetal**  **Genotype** | **Paternally-transmitted**  **Allele** | **Maternally-transmitted**  **Allele** |
| --- | --- | --- | --- |
| A/A | A/A | A | A |
| A/a | A/A | A | A |
| A/a | A/a | uninformative | uninformative |
| a/a | A/a | A | a |
| A/A | A/a | a | A |
| A/a | a/a | a | a |
| a/a | a/a | a | a |
