# Supplementary material for: Associations between a fetal imprinted gene allele score and late pregnancy maternal glucose concentrations
Source: Diabetes Metab. 2017 Sep;43(4):323–31. doi: 10.1016/j.diabet.2017.03.002 (PMC5507297; doi:10.1016/j.diabet.2017.03.002)
Supplement: Supplementary file 3 [file mmc3.docx]

**Table S3:** *P*-values of the associations between the fetal imprinted gene SNP alleles that make up the composite score and maternal glucose concentration z-scores one after the oral consumption of a glucose load in the Cambridge Baby Growth Study and the Cambridge Wellbeing Study.

|  |  | **Cambridge Baby Growth Study**  **(n = 511̴ 526)** | | | **Cambridge Wellbeing Study**  **(n = 253 ̴ 273)** | | |
| --- | --- | --- | --- | --- | --- | --- | --- |
| **SNP** | **Transmission to Fetus** | **Non-Risk Allele** | **Risk Allele** | ***P*-value** | **Non-Risk Allele** | **Risk Allele** | ***P* -value** |
| rs10770125 | Paternal | -0.074  (-0.193, 0.045) | 0.135  (0.020, 0.249) | 0.013 | -0.223  (-0.404, -0.042) | 0.143  (-0.032, 0.318) | 4.5 x 10^-3^ |
| rs2585 | Paternal | -0.043  (-0.135, 0.050) | 0.355  (0.163, 0.528) | 2.8 x 10^-4^ | -0.118  (-0.257, 0.219) | -0.035  (-0.290, 0.219) | 0.5 |
| rs231841 | Maternal | -0.013  (-0.114, 0.089) | 0.090  (-0.065, 0.245) | 0.3 | -0.094  (-0.239, 0.052) | 0.184  (-0.050, 0.418) | 0.048 |
| rs7929804 | Maternal | -0.046  (-0.172, 0.081) | 0.064  (-0.051, 0.179) | 0.2 | -0.104  (-0.288, 0.079) | 0.077  (-0.091, 0.246) | 0.2 |

Data are mean (95 % confidence interval) z-scores.
