# Supplementary material for: Associations between a fetal imprinted gene allele score and late pregnancy maternal glucose concentrations
Source: Diabetes Metab. 2017 Sep;43(4):323–31. doi: 10.1016/j.diabet.2017.03.002 (PMC5507297; doi:10.1016/j.diabet.2017.03.002)
Supplement: Supplementary file 4 [file mmc4.docx]

**Table S4:** *P*-values of the associations between the composite fetal allele score (minus the effects of paternally-transmitted fetal *IGF2* rs10770125 and rs2585 individually) and maternal glucose concentration z-scores one after the oral consumption of a glucose load in the Cambridge Baby Growth Study and the Cambridge Wellbeing Studies combined.

| Fetal Gene Allele Score | n | *P*-value | Adjusted r^2^ |
| --- | --- | --- | --- |
| Full composite fetal allele score | 981 | 4.3 x 10^-6^ | 2.0 |
| Full composite fetal allele score minus the effect of paternally-transmitted fetal rs10770125 | 981 | 2.0 x 10^-5^ | 1.7 |
| Full composite fetal allele score minus the effect of paternally-transmitted fetal rs2585 | 981 | 4.5 x 10^-5^ | 1.6 |
