# Supplementary material for: Associations between a fetal imprinted gene allele score and late pregnancy maternal glucose concentrations
Source: Diabetes Metab. 2017 Sep;43(4):323–31. doi: 10.1016/j.diabet.2017.03.002 (PMC5507297; doi:10.1016/j.diabet.2017.03.002)
Supplement: Supplementary file 5 [file mmc5.docx]

**Table S5:** Results of tests for heterogeneity of the meta-analyses.

| Meta-Analysis | Q | Degrees of Freedom | *P*-value | I^2^ (%) |
| --- | --- | --- | --- | --- |
| Paternally-transmitted fetal rs2585 | 2.57 | 2 | 0.28 | 22.31  (0.00, 97.39) |
| Paternally-transmitted fetal rs10770125 | 1.50 | 2 | 0.47 | 0.00  (0.00, 95.54) |
| Maternally-transmitted fetal rs231841 | 1.68 | 2 | 0.43 | 0.00  (0.00, 96.02) |
| Maternally-transmitted fetal rs7929804 | 0.19 | 2 | 0.91 | 0.00  (0.00, 64.89) |
| Composite Fetal Imprinted Gene Allele Score | 1.20 | 2 | 0.55 | 0.00  (0.00, 0.00) |

Data are mean (95 % confidence interval) where specified.
