# Supplementary material for: Associations between a fetal imprinted gene allele score and late pregnancy maternal glucose concentrations
Source: Diabetes Metab. 2017 Sep;43(4):323–31. doi: 10.1016/j.diabet.2017.03.002 (PMC5507297; doi:10.1016/j.diabet.2017.03.002)
Supplement: Supplementary file 6 [file mmc6.doc]

**Table S6:**

The associations between placental *KCNQ1* and *KCNQ1OT1* expression at term and maternally-transmitted fetal *KCNQ1/KCNQ1OT1* alleles in the Cambridge Baby Growth Study for the SNPs that were included in the composite fetal allele score.

|  | *Placental KCNQ1 expression* | | | | *Placental KCNQ1OT1 expression* | | | |
| --- | --- | --- | --- | --- | --- | --- | --- | --- |
| Maternally-transmitted  Fetal SNP | Non-Risk Allele | Risk Allele | *P*-value | Effect Size  (Cohen’s d) | Non-Risk Allele | Risk Allele | *P* -value | Effect Size  (Cohen’s d) |
| rs231841 | 0.074  (0.066, 0.082)  (n = 14) | 0.089  (0.077, 0.100)  (n = 7) | 0.04 | 0.42 | 0.007  (0.005, 0.009)  (n = 14) | 0.005  (0.002, 0.008)  (n = 7) | 0.16 | 0.17 |
| rs7929804 | 0.078  (0.065, 0.091)  (n = 10) | 0.080  (0.067, 0.092)  (n = 10) | 0.88 | 0.11 | 0.007  (0.005, 009)  (n = 10) | 0.005  (0.003, 0.007)  (n = 10) | 0.32 | 0.16 |

Data are mean (95 % confidence interval). The risk allele refers to the allele associated with higher maternal glucose concentrations in late pregnancy.
