# Supplementary material for: Associations between a fetal imprinted gene allele score and late pregnancy maternal glucose concentrations
Source: Diabetes Metab. 2017 Sep;43(4):323–31. doi: 10.1016/j.diabet.2017.03.002 (PMC5507297; doi:10.1016/j.diabet.2017.03.002)
Supplement: Supplementary file 7 [file mmc7.docx]

**APPENDIX: Supplementary Methods**

*Placental KCNQ1(OT1) Gene Expression*

Although the genes that were tested are all paternally-expressed at some point in development those SNPs selected to tag *KCNQ1OT1* could also partially tag the maternally-expressed imprinted gene *KCNQ1* because of its close proximity (the imprinting control region of the *KCNQ1* cluster accommodating the *KCNQ1OT1* promoter). We therefore measured placental *KCNQ1OT1* and *KCNQ1* expression at term and related these to *KCNQ1OT1* SNPs that were significantly associated with maternal glucose concentrations to inform which of the two genes may underpin these associations.

420 term Cambridge Baby Growth Study placentas were collected and processed within an hour of delivery, snap frozen, and then stored at -20°C (1~2 years) until samples were removed for RNA extraction. A total of 72 placentas were initially studied due to having fetal *KCNQ1OT1* SNP alleles available. From these, while keeping the tissue frozen, three samples weighing approximately 10 mg each were macrodissected from the basal plate of each placenta and the outer 3 mm of each discarded. Samples were then pooled before homogenization and extraction using Qiagen Tissueruptor and RNeasy fibrous mini kits (Qiagen Ltd., Manchester, U.K.) run as per the manufacturer’s instructions, including the use of the DNase I that was included in the kit. RNA was quantified and its purity assessed (A260/A280) using a Nanodrop v. 1.0 (Labtech Ltd., Ringmer, U.K.). The integrity was assessed using an Agilent Bioanalyzer (Agilent Technologies, Stockport, U.K.). Only samples with a 260/280 nm absorbance ratio > 2.0 and an integrity score > 6.4 were used (n = 21). Extracted RNA samples were stored in RNAsecure (Life Technologies Ltd., Paisley, U.K.) at -80°C until analysis.

Synthesis of cDNA was performed using Bioline SensiFAST cDNA Synthesis Kits (London, U.K.) according to the manufacturer’s instructions (using 1 µg RNA in a total reaction volume of 20 µL, reverse transcriptase and random hexamers, and incubating at 42°C for 30 min.). TaqMan Gene expression Assays (Life Technologies) were purchased for *KCNQ1* (Hs00923522_m1; lot no. 1243638; 57 bp amplicon) and *KCNQ1OT1* (Hs03665990_s1; lot no. P150520-002 B03; 101 bp amplicon) transcripts and the validated placental reference genes *YWHAZ* (Hs03044281_g1; lot no. 1386980; 106 bp amplicon), *TOP1* (Hs00243257_m1; lot no. 1370623; 101 bp amplicon), and *UBC* (Hs01871556_s1; lot no. 1328713; 135 bp amplicon) (Cleal JK et al. (2009) Placenta 30:1002-1003). The quantitative PCR reactions were performed using 100 ng cDNA per reaction plus 10 µL TaqMan Universal Master Mix (Life Technologies), 1 µL each assay mix and RNase-free water to make the total reaction volume 20 µL. The amplification was run on an Agilent Technologies AriaMx Real-Time PCR system according to the manufacturer’s instructions with one cycle of 50^o^C for 2 min. and 95^o^C for 10 min., followed by 40 cycles of 95^o^C for 15 sec. and 60^o^C for 1 min. Data was initially processed using Agilent AriaMx software version 1.0.

The placental *KCNQ1* and *KCNQ1OT1* expression values (adjusted for the expression of the reference genes) were grouped according to the maternally-transmitted fetal rs231841 or rs7929804 alleles and analysed using the comparative C_T_ method included in RT^2^ Profiler PCR Array Data Analysis version 3.5 (available at <http://pcrdataanalysis.sabiosciences.com/pcr/arrayanalysis.php>; accessed 05 February 2016).
